# Ophthalmic Manifestation and Pathological Features in a Cohort of Patients With Linear Nevus Sebaceous Syndrome and Encephalocraniocutaneous Lipomatosis

**DOI:** 10.3389/fped.2021.678296

**Published:** 2021-05-20

**Authors:** Yan Yan, Siyi Zhang, Henghua Zhou, Yixiong Zhou, Yao Fu

**Affiliations:** ^1^Department of Ophthalmology, Shanghai 9th Peoples Hospital Affiliated to Shanghai Jiaotong University School of Medicine, Shanghai, China; ^2^Shanghai Key Laboratory of Orbital Diseases and Ocular Oncology, Shanghai, China; ^3^Department of Pathology, Shanghai 9th Peoples Hospital Affiliated to Shanghai Jiaotong University School of Medicine, Shanghai, China

**Keywords:** encephalocraniocutaneous lipomatosis, linear nevus sebaceous syndrome, oculoectodermal syndrome, ophthalmic symptoms, pathological features

## Abstract

**Purpose:** This study aimed to figure out the association between ophthalmic and pathological features in patients with Linear Nevus Sebaceous Syndrome (LNSS) and in patients with Oculoectodermal Syndrome—Encephalocraniocutaneous Lipomatosis (OES-ECCL).

**Methods:** It is a retrospective, non-consecutive, observational case series. Twenty-seven patients (12 with LNSS and 15 with OES-ECCL, 41 eyes) referred to the Department of Ophthalmology of the Shanghai Ninth People's Hospital between 2000 and 2020 were included. The mean age of the study population for the first-time consult was 5.7 years, ranging from 3 months to 34 years. Clinical notes, pathological records, and imaging findings were reviewed in all the patients.

**Results:** Fourteen (51.9%) cases showed bilateral ocular involvement. Epibulbar choristomas were seen in all the patients. All the lesions involved the conjunctiva and cornea simultaneously. Multiple lesions were observed in 12 eyes. Of the 14 excised lesions, 11 were found to be complex choristomas. Further, 24 (89%) patients had eyelid coloboma. Also, 13 patients (48%) were diagnosed with strabismus, and 12 patients (44%) had abnormal fundus imaging, including optic nerve hypoplasia.

**Conclusions:** LNSS and OES-ECCL shared common ophthalmic features, including epibulbar choristomas with distinctive characteristics, eyelid coloboma, strabismus, and optic nerve hypoplasia. The complex choristoma was found to be associated with the diseases. These specific patterns can be diagnostic clues to distinguish them from other syndromes, such as craniofacial defects, and to remind ophthalmologists that such patients require additional dermatological and neurological examinations and referral. Moreover, a thorough evaluation of ocular conditions is imperative for early interventions.

## Introduction

Linear Nevus Sebaceous Syndrome (LNSS) and Oculoectodermal Syndrome—Encephalocraniocutaneous Lipomatosis (OES-ECCL) are unique developmental mosaic RASpathies of phacomatoses (neurocutaneous disorders), encompassing both distinctive features and a broad spectrum of manifestations (1–3). Nevus sebaceous is a hallmark of LNSS, characterized as papillomatous hyperplasia of the epidermis (4). LNSS was first reported by Schimmelpenning in 1957 (5). And in 1962 Feuerstein and Mims reported it as a triad of linear naevus sebaceous with epilepsy and developmental delay (6). Several names have been applied since so far, such as epidermal nevus syndrome, Schimmelpenning Feufstein-Mims syndrome, Solomon syndrome and Jadassohn's syndrome. In 1975, Solomon and Esterly reviewed 60 cases and broadened the spectrum of multiorgan involvement: ophthalmological, neurologic, skeletal, cardiovascular, and urologic comorbidities (4). The incidence of ocular Involvements is up to around 60%, and clinical presentations vary from each other, of which the main abnormalities are strabismus, colobomas and choristomas. Complex choristoma is the most characteristic but not the specific ocular feature of the syndrome. Other rare conditions include iris and chorioretinal colobomas, generalized retinal degeneration, antimongoloid lid fissures, asymmetry of orbital bones, circumscribed choroidal hemangioma, etc. (7, 8).

Encephalocraniocutaneous lipomatosis (ECCL), also called Haberland syndrome or Fishman's syndrome, was first reported by Haberland in 1970 (9). There have been roughly 75 cases reported till now. The most characteristic anomalies are nevus psiloliparus (hairless fatty tissue nevus of the scalp), epibulbar choristomas and ipsilateral central nervous system (CNS) anomalies (10). The clinical phenotype of CNS anomalies comprises cranial lipomas, arachnoid cysts, seizures and intellectual disability (11, 12). The ocular manifestations had many similarities in ECCL and LNSS, which include choristomas, coloboma and periocular skin tag. Choristomas, with or without other associated ocular anomalies are the most common clinical features, which can be seen in approximately 80% of the patients. Lipodermoids are usually mild and won't affect the optic axis as well as visual development. However, dermolipomas always present with other atypical ophthalmic malformations, including anterior segment anomalies, cornea anomalies, ocular coloboma, globe calcifications, etc. The pattern of eye findings in ECCL patients was found to be very consistent and recognizable (13).

Oculoectodermal syndrome was first described in 1993 by Toriello et al. (14). Less than 20 cases have been recorded since then (2, 13, 15). Classic clinical features include the association of epibulbar dermoids and aplasia cutis congenita (16). Nevertheless the epibulbar dermoids are not the distinct findings, which can also occur in Goldenhar syndrome. Additional ocular involvements include strabismus, microcornea, nystagmus, etc. The clinical manifestations of the overlap between OES and ECCL are observable with regard to ocular and dermatological symptoms. Evidence showed that the OES is a mild variant of ECCL, differing primarily by lack of intracranial anomalies on brain imaging (17).

The great overlap in clinical manifestations makes it hard to distinguish them from other neurocutaneous disorders.The incidence of ophthalmic anomalies of LNSS and OES-ECCL was around 60–80%, which was relatively high (4, 7, 13, 16, 18). Several ophthalmic conditions have been reported as a part of the case review, which were found to be common between the syndromes, including ocular colobomas and choristomas. Besides, various rare ocular symptoms can also be seen (8, 11, 16, 18). Moreover, the common symptoms are not specific and also can be seen in other diseases as craniofacial defects (4, 13, 18, 19), which makes, leading pediatricians and ophthalmologists to be easily confused with the conditions and overlook the extraocular symptoms. However, few studies explored the association between ocular manifestations in LNSS and OES-ECCL owing to the low incidence of diseases; even few ophthalmologists had the experience of dealing with multiple patients. Whether each of the syndromes has specific ocular manifestations for differential diagnosis or great clinical ophthalmic overlap, like the pattern of skin or neurological symptoms, was still unclear.

This study was performed to share the experience of 27 patients (41 eyes) with LNSS and OES-ECCL. Attempts were made to find distinct clinical patterns and illustrate the common ground and variants among the syndromes by reviewing all the ocular manifestations and pathological findings.

## Method

This study was conducted in compliance with the tenets of the Declaration of Helsinki and approved by the ethics committee of the Shanghai Ninth People's Hospital (approvals ID: S9H9-2019-T359-1). The study was a retrospective, non-consecutive, observational case series. All the 27 patients (41 eyes) diagnosed with LNSS or OES-ECCL, including LNSS (12 patients, 20 eyes) and OES-ECCL (15 patients, 21 eyes; OES 10 patients, 14 eyes; ECCL five patients, seven eyes), were referred to the Department of Ophthalmology of the Shanghai Ninth People's Hospital between January 2000 and January 2020. Clinical notes, pathological records, and imaging findings were reviewed in detail. A full ophthalmologic evaluation was performed with special attention to the eyelid coloboma, epibulbar choristomas, eyelid tags, retinal hypoplasia, and strabismus. The assessment included measurement of a slit-lamp biomicroscopic examination, fundus examination, ultrasound biomicroscopy, B-scan ultrasonography, optical coherence tomography, and three-dimensional orbital computed tomography scan for bony defects. One patient underwent targeted next-generation sequencing of skin lesions. Eleven patients underwent excision of epibulbar choristomas. Pathologic diagnoses were confirmed by two pathologists. All patients were photographed. Informed consent forms were signed by the patients and guardians of the children.

## Results

### Study Populations

**Figure 1:**
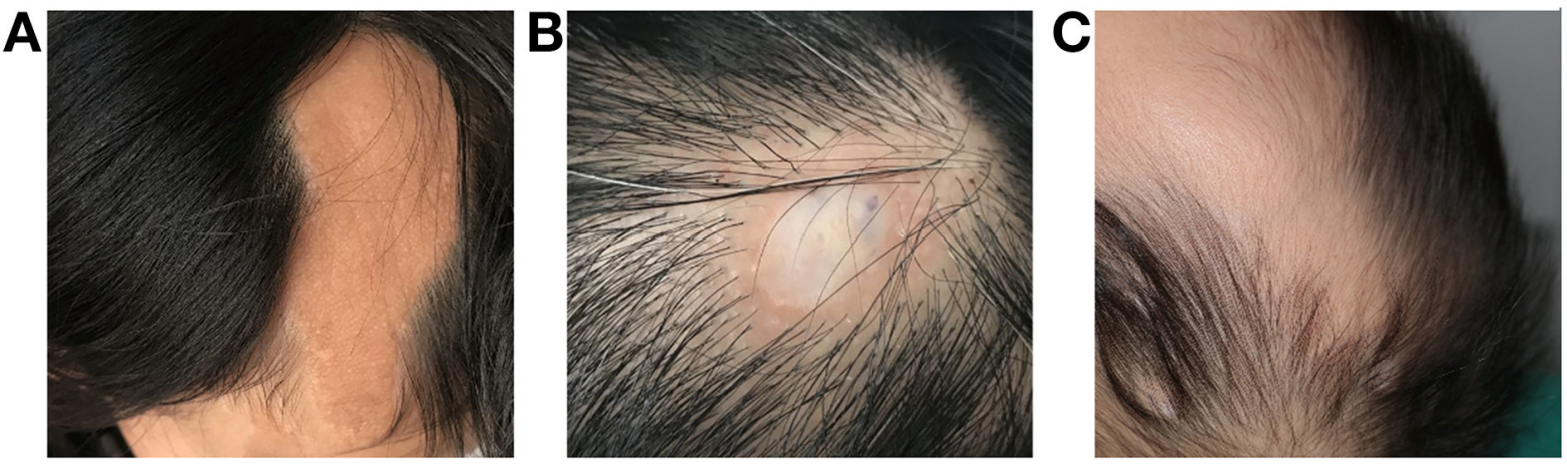
Dermatological manifestations of patients with LNSS an OES-ECCL. Sebaceous nevus in a patient with LNSS (patient 4) **(A)**. Nevus psiloliparus (ECCL, patient 25) **(B)** and congenital alopecia (OES, patient 13) **(C)**.

A total of 27 patients with neurocutaneous syndromes were identified from the clinical database of the Shanghai Ninth People's Hospital, of which 12 were diagnosed with LNSS and 15 with OES-ECCL (10 with OES and five with ECCL) (Figure 1). The mean age of the study population for the first-time consultation was 5.7 years, ranging from 3 months to 34 years. No marked sexual dominance was found in patients with a ratio of 1:1 in female vs. male participants. Patient 3 showed a low-level heterozygous mutation in the KRAS gene (c.35C>T; p.G12D, 5 %) (20). Systemic symptoms are listed in Table 1.

**Table 1:** Basic information and systemic manifestations of the patients.

**Diagnosis**		**Patient no**.	**Sex**	**Age**	**Skin**			**Neurology**			
					**Nevus sebaceous**	**Nevus psiloliparus**	**Aplasia cutis congenita**	**Intellectual disability**	**Seizure**	**Other**	**Other systemic involvement**
LNSS		1	F	8 years	+						
		2	M	4 months	+						
		3^*^	F	1 year	+			+			
		4	F	1 year	+						G, H, and I
		5	M	7 years	+						
		6	M	5 years	+			+			
		7	F	32 years	+						
		8	F	6 years	+			+	+		
		9	F	3 years	+						
		10	F	11 years	+						
		11	M	3 years	+						
		12	M	1 year	+			+		A	J, K, L, M, and N
OES-ECCL	OES	13	M	7 months			+				
		14	F	5 years			+				
		15	M	3 years			+				O
		16	M	7 years			+				P
		17	F	1 year			+				
		18	F	6 years			+				
		19	F	1 year			+				
		20	F	12 years			+				
		21	F	34 years			+				
		22	M	10 months			+				
	ECCL	23	M	1 year						B	G
		24	M	1 year		+			+		
		25	M	4 years		+			+	A and C	
		26	M	5 months		+		+	+	A, C, D, and E	O and Q
		27	M	3 months		+				C, E, and F	

*A, Ventricular dilation; B, pontine lipoma; C, arachnoid membrane tumor; D, pachygyria; E, cerebral hemorrhage; F, hypoxic ischemic encephalopathy; G, cleft palate; H, anotia; I, atresia aural; J, atrial septal defect; K, patent ductus arteriosus; L, pulmonary arterial hypertension; M, neonate hyperbilirubinemia; N, hydrocele of tunica vaginalis; O, temporal bone defect; P, hypoesthesia of limb (right); Q, atrial septal aneurysm*.

* *Patient showed a KRAS somatic mosaic mutation*.

### Ophthalmic Manifestations

**Figure 2:**
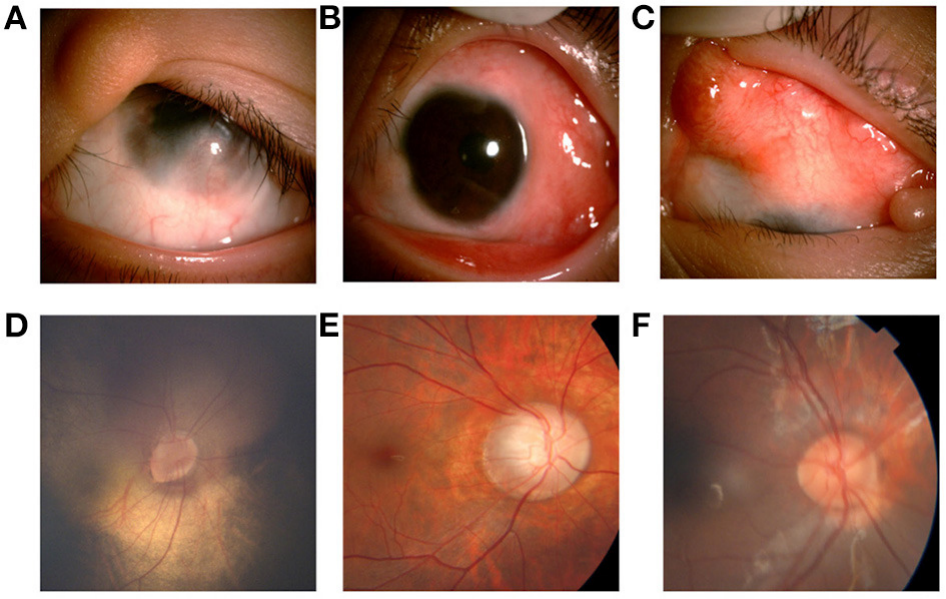
Ocular manifestations of patients with LNSS an OES-ECCL. Single flat epibulbar choristoma involving the cornea with unclear boundaries and upper eyelid coloboma with skin tag (LNSS, patient 9) **(A)**. Mutiple epibulbar choristomas involving the cornea, and esotropia (LNSS, patient 1) **(B)**. Large, raised epibulbar choristoma, and upper eyelid coloboma (ECCL, patient 25) **(C)**. The oval optic disc, inferior temporal optic disc coloboma, and the vessels crawl out the edge of the disc, which is similar to the pattern of morning glory disc. The patient with corneal choristoma was examined under anesthesia (LNSS, patient 3) **(D)**. The boundary of the optic disk is not clear with a lighter color, and the exposed scleral ring can be seen around the disk (ECCL, patient 25) **(E)**. Congenital coloboma of the optic disk (OES, patient 14) **(F)**.

The ocular symptoms are listed in Table 2. Among all the participants, 14 (51.9%) showed bilateral ocular involvement. Choristomas, as benign epibulbar tumors, were seen in all the patients (41/41 eyes). Multiple lesions (Figure 2B) were seen in 12 eyes. All the lesions, including eight limbus lesions, covered both the conjunctiva and the cornea simultaneously. More interestingly, one of the lesions spanned the bulbar conjunctiva and the outer canthus. Except for isolated limbus lesions, the distribution of the bulbar conjunctiva choristomas (38 eyes) included two choristomas in ≤ 1 quadrant, 10 in more than 1 but ≤ 2 quadrants, 16 in more than 2 but ≤ 3 quadrants (42%), and 10 in more than 3 quadrants (26%). The most common lesion location was superotemporal quadrant (38/38 eyes, 100%) and infratemporal quadrant (28/38 eyes, 74%). Five of the patients had symblepharon secondary to choristomas.

**Table 2:** Ocular manifestations of patients with LNSS and OES-ECCL.

**Diagnosis**		**Patient no**.	**Bilateral**	**Choristoma**	**Ptosis**	**Eyelid coloboma**	**Strabismus**	**Eyelid tag**	**Optic nerve abnormalities**	**Symblepharon**	**Other symptoms**
LNSS		1	+	+	+	+	+	+	+		a
		2		+					+		b
		3	+	+	+	+	+	+	+	+	
		4	+	+	+	+		+			c
		5	+	+	+	+	+				d
		6	+	+	+	+					
		7		+	+	+					
		8		+	+	+	+	+	+		d, e, and f
		9		+		+		+	+		
		10	+	+	+	+	+	+		+	
		11	+	+	+	+	+	+	+		e and g
		12	+	+	+	+	+	+	+	+	e
OES-ECCL	OES	13		+		+	+	+			
		14		+					+		
		15	+	+		+					e
		16	+	+	+	+	+				h
		17	+	+	+	+				+	h
		18		+		+		+			
		19	+	+		+	+				i
		20		+		+		+			e
		21		+		+					
		22		+	+	+	+	+	+		
	ECCL	23	+	+		+		+	+		j
		24		+		+	+		+		b, k, and h
		25	+	+	+	+	+	+	+	+	l
		26		+				+			
		27		+		+		+			

*a, Retinal anomalies; b, corneal neovascularization; c, vitreous opacity; d, microphthalmia; e, trichiasis; f, entropion; g, thinning of the retina; h, cornea opacity; i, lower eyelid retraction; j, coloboma chorioideae; k, A-R syndrome; l, globe calcification*.

Eyelid anomalies were the second common conditions. Further, 24 children (89%) had eyelid coloboma (Figures 2A,C), of which 15 (63%) had an accessory skin tag (Figure 2A). The defects mostly affected the middle and inner one third of the upper eyelid. Also, 14 patients (51.9%) had ptosis. Lid abnormality led to trichiasis in five patients (18.5%). Moreover, 13 patients (16 eyes, 48%) had strabismus. Esotropia was found in eight eyes, catatropia in five eyes, esocataphoria in one eye, exocataphoria in one eye, and exotropia in one eye. Moreover, 12 patients (44%) had abnormal fundus imaging showing optic nerve abnormalities. Optic nerve hypoplasia (Figures 2D,E) was found in nine patients.

**Figure 3:**
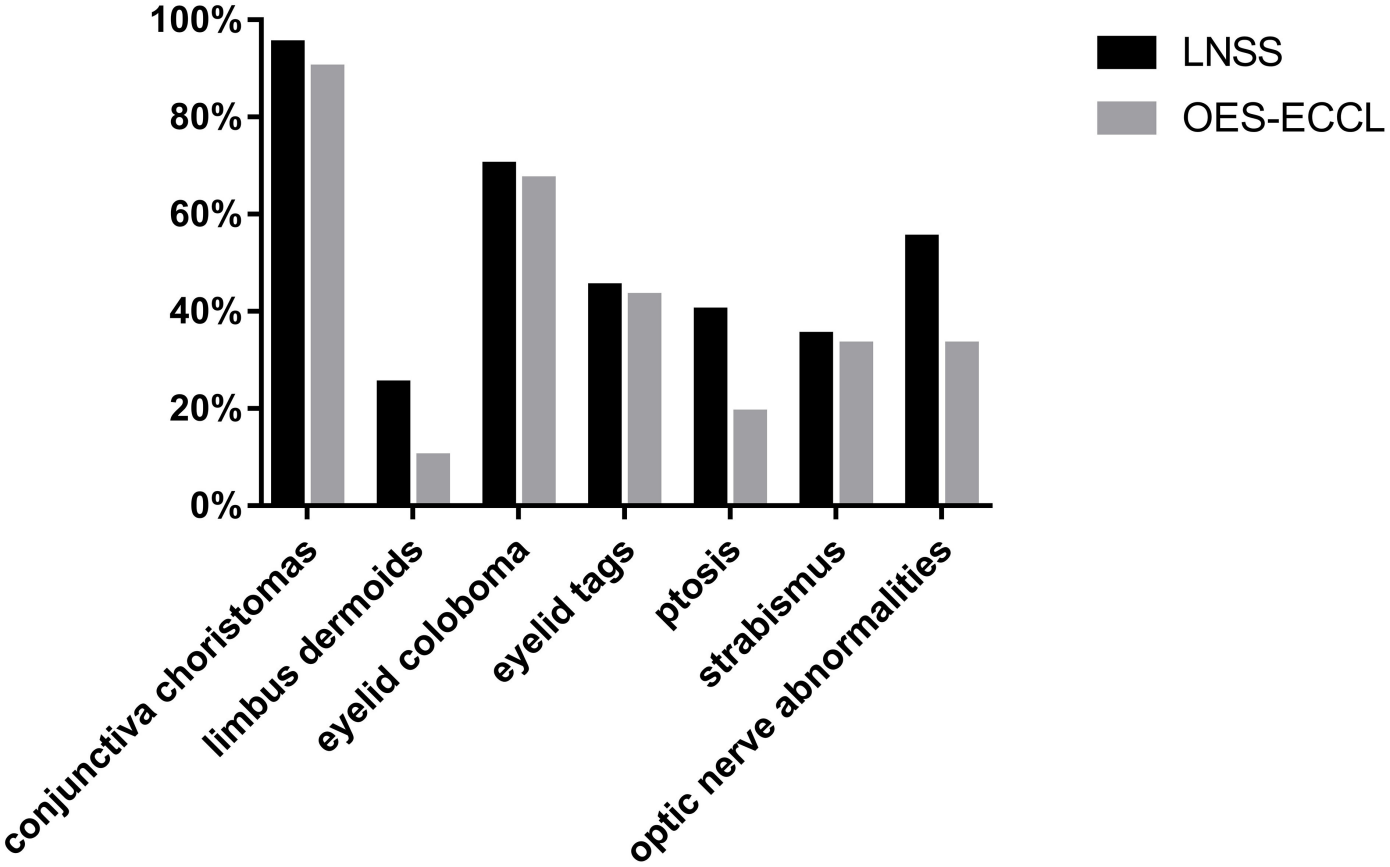
The prevalence of main ocular manifestations in patients with LNSS and OES-ECCL.

Other rare malformations included corneal opacity, lower eyelid retraction, coloboma chorioideae, thinning retina, and enlarged C/D ratio. The severity of skin and eye findings was not always consistent. The prevalence of symptoms is presented in Figure 3.

### Pathology

Lesions on conjunctiva varied in gross appearance, ranging from small, flat pterygium-like lesions to large raised masses. However, unclear boundaries and the involvement of the cornea were common among most lesions.

**Figure 4:**
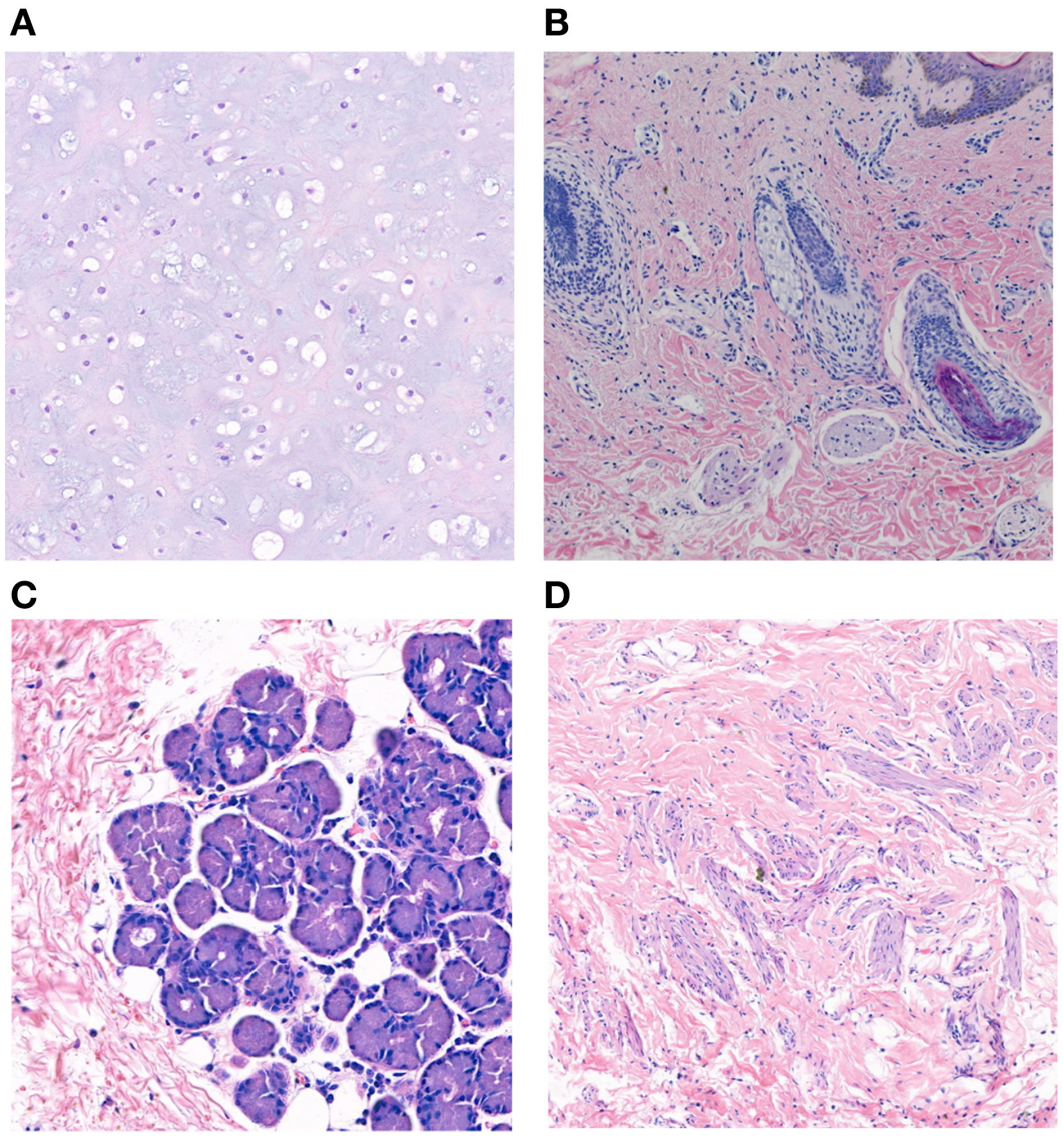
Histopathological studies of complex choristoma cases. The complex choristoma was composed of cartilaginous tissue (patient 7) **(A)**. Immature hair follicle tissues combined with sebaceous glands were found in the biopsy of conjunctival lesions (patient 24) **(B)**. The complex choristoma contained lacrimal gland tissue (patient 9) **(C)**. The large complex choristoma contained both fibrous connective tissue and smooth muscle (patient 11) **(D)** (hematoxylin-eosin-stained, ×20 original magnification).

Twelve patients with choristomas underwent surgical removal. The histopathological findings are summarized in Table 3. Two patients had dermolipomas, another one was diagnosed with limubs dermoid, whereas the remaining (78.5%) had complex choristomas (Figure 4). Complex choristomas composed of fibrous connective tissue, smooth muscle, and glandular tissue, such as lacrimal tissue and sebaceous glands, accounted for eight cases. Hyaline cartilaginous tissues were found in five cases.

**Table 3:** Histopathologic features of epibulbar complex choristoma in 12 surgically resected cases.

**Diagnosis**		**Patients**	**Lesion**	**Location (quadrant)**	**Fibrous connective tissue**	**Hair follicle tissue**	**Adipose tissue**	**Lacrimal gland**	**Epidermal tissue**	**Sebaceous gland**	**Smooth muscle bundles**	**Cartilage**
LNSS		4	Complex choristoma OD	Temporal with eyelid involvement	+	+				+		
			Complex choristoma OS	Temporal	+		+	+	+			+
		7	Complex choristoma	Upper	+		+				+	+
		9	Complex choristoma	Upper	+	+	+	+	+	+	+	
		11	Complex choristoma	Circumferential	+	+	+	+	+	+	+	
			dermoid	limbus	+	+				+		
		12	Complex choristoma	Upper	+					+	+	+
OES-ECCL	OES	14	Complex choristoma	Upper	+		+	+				+
		16	Dermolipoma	Upper	+		+					
		19	Complex choristoma	Upper with temporal	+		+	+	+		+	
		22	Complex choristoma	Temporal	+	+	+		+	+		+
	ECCL	24	Complex choristoma	Temporal	+	+	+	+		+		
		25	Complex choristoma	Circumferential	+		+					+
		26	Dermolipoma	Upper	+		+					

## Discussion

In this study, the largest case series was used to study the association between ocular manifestations in LNSS and OES-ECCL. No obvious difference was found with regard to the ocular symptoms between the two groups, which was in contrast with the pattern of neurological and dermatological symptoms (Figure 3). Besides, shared features included epibulbar choristomas, eyelid colobomas, periocular skin tags, and optic nerve anomalies. The ocular symptoms might also follow a certain pattern according to clinical experience; patients with epibulbar choristomas involving both conjunctiva and cornea, combined with eyelid coloboma, might lead to the suspicion of having LNSS or OES-ECCL. Of these, patients with ECCL were more likely to have eyelid skin tags (74%) on account of the pathogenesis. The complex choristomas were both closely associated with LNSS (six in seven lesions) and OES-ECCL (five in seven lesions).

Epibulbar choristomas (except for limbus dermoids) were mostly observed in the present cohort (38/41 eyes, 92.7%). Epibulbar choristomas are relatively rare benign tumors. They are masses of histologically normal tissue in an abnormal location, mostly located on the limbus or solely on the superior temporal quadrant of conjunctiva, with clear boundaries (19, 21). In this cohort, a type of epibulbar choristomas with unique features was identified, which were large masses involving both the cornea and the conjunctiva, with unclear boundaries. The unique characteristics may distinguish them from dermoids, lipodermoids, and prolapsed orbital fat. Most of them (26/38 eyes) filled in more than two quadrants of the bulbar conjunctiva in this study, and all covered the supratemporal quadrant. All masses were reported without growth in size by the parents.

For associated ocular anomalies, eyelid coloboma is the most recorded anomaly with a featured location of the upper inner one-third eyelid. Periocular skin tags, located on the site of eyelid coloboma, are also common among patients. Strabismus was one of the common conditions with the foremost diagnosis of esotropia. The etiology of strabismus in children is complicated. The possible causes include congenital abnormalities of extraocular muscle development, nuclei abnormalities that dominate eye movements, abnormal vision development, and restrictive diseases. Patients with strabismus also have large choristomas, mostly combined with ptosis, optic nerve abnormalities, and symblepharon, attributable to multiple causes. Large-sized choristomas and secondary symblepharon may cause abnormal eye positions and restricted eye movement. At the same time, strabismus of visual deprivation occurs when patients have abnormal development of the optic nerve, severe ptosis, and blocked visual axis. When dealing with particular patients, the first step is to remove the restrictive factors and the obstructions that affect the development of the optic axis, and then re-evaluate the strabismus and the fundus. The principle of the treatment is to deal with amblyopia to improve vision.

Fundus anomalies are hard to describe in a predictable pattern due to significant clinical variability (22). However, coloboma is the most frequent malformation involving the optic disk (Figure 2F) (13, 23). Optic disk hypoplasia was found in most cases, appearing typically as a white, decentered excavation of the optic disk, or with marginal hypoplasia, accompanied by surrounding retina-choroidal atrophy. Congenital disk conformation anomalies can be related to all levels of vision, ranging from 20/200 to finger counting (24). Thus, fundus assessments should be emphasized for routine screening for patients as early as possible.

Regarding the histological diagnosis, complex choristomas (11/14 lesions) are associated with the subtypes of a neurocutaneous syndrome. Complex choristomas contain cells from two or more different tissue types, such as bone, cartilage, nerve, muscle, sebaceous secretions, or lacrimal tissues. Complex choristomas can be sporadic cases or can be associated with Goldenhar syndrome and LNSS (25–28). For patient 24, the first biopsy of the nasal part showed a dermolipoma, and the result of the second biopsy of the temporal part disclosed a complex choristoma with immature hair follicle tissue, sebaceous gland, and hyperplasia of adipose tissue. Thus, it is suggested that biopsy at more than one site should be taken into consideration for large epibulbar masses associated with congenital neurocutaneous syndromes.

LNSS and OES-ECCL are the results of postzygotic KRAS, HRAS, NRAS, and FGFR1 mutations (2, 29). The genetic testing of somatic mutations in KRAS and FGFR1 genes could provide a tool for early diagnosis. In DNA from the skin lesion of patient 3, mosaicism of KRAS c.35C>T; p.G12D was found. The mutation identified in this study was also reported in a patient with rhabdomyosarcoma (19, 30) which supports the conclusion of emerging mutation overlap between mosaic developmental disorders and tumorigenesis (29). Even with relatively large sample sizes, the rarity of the diseases might limit the analysis. Also, fundus characteristics were hardly summarized thoroughly because the evaluation was impossible for most of the patients with corneal anomalies. The RAS-MAPK pathway regulates important cellular processes, including DNA synthesis. Therefore, further functional studies of the mutation spot should be carried out (29). Despite these limitations, the study provided a fresh perspective on the association between ocular manifestations in LNSS and OES-ECCL. Unlike the cutaneous or neural symptoms, these types of diseases have no distinct clinical patterns of ocular anomalies. In other words, a differential diagnosis cannot be made based solely on ophthalmic examinations. However, these diseases have common but unique ophthalmic features, such as distinct epibulbar choristomas, or combinations of choristomas with eyelid colobomas. These specific patterns can be diagnostic clues to distinguish them from other syndromes, such as craniofacial defects and Goldenhar syndrome, and to remind ophthalmologists that such patients require additional dermatological and neurological examinations and referral. The ocular anomalies can be primary clinical presentations, requiring more attention.

The ophthalmic referral is vital as early as possible for patients who may only have a suspected diagnosis. Eyelid coloboma is with high incidence in these two diseases. It is proved that early surgical treatment of eyelid coloboma can not only improve the transparency of the cornea and the homeostasis of the ocular surface but also protect the visual development. Timely treatment requires a multidisciplinary physician collaboration. For pediatricians, understanding the characteristics of eye complications for timely referral can improve the patient's appearance and protect visual development, which reduces the burden on the children's family and improves their quality of life. Updating research on these rare congenital neurocutaneous syndromes' clinical features and experience is a win-win scenario for both patients and doctors.

## Conclusions

The ocular involvement of LNSS and OES-ECCL has a great clinical overlap, including epibulbar choristomas, eyelid coloboma, strabismus, and optic nerve hypoplasia. The distinctive characteristic is a large epibulbar complex choristoma involving the cornea and conjunctiva, with unclear boundaries. The unique clinical patterns can serve as diagnostic clues to differ LNSS and OES-ECCL from other syndromes. A thorough evaluation of ocular conditions is imperative for early interventions.

## Data Availability Statement

The original contributions generated for this study are included in the article/supplementary material, further inquiries can be directed to the corresponding author/s.

## Ethics Statement

The studies involving human participants were reviewed and approved by the Ethics Committee of Shanghai Ninth People's Hospital and complied with the tenets of the Declaration of Helsinki for clinical research (IRB: S9H9-2019-T359-1). Written informed consent to participate in this study was provided by the participants' legal guardian/next of kin.

## Author Contributions

YY, YF, and YZ had full access to all of the clinical data in the study and take responsibility for the integrity of the data and the accuracy of the case series. YY and YF: study concept and design. YY, HZ, SZ, YZ, and YF: acquisition, analysis of data. YY: drafting of the manuscript. YY, YZ, and YF: critical revision of the manuscript for important intellectual content. YF: obtained funding, administrative, technical, or material support. YZ and YF: study supervision. All authors have read and approved the manuscript and agree to be accountable for all aspects of the work.

## Conflict of Interest

The authors declare that the research was conducted in the absence of any commercial or financial relationships that could be construed as a potential conflict of interest.

## Abbreviations

LNSS: linear nevus sebaceous syndrome
OES: oculoectodermal syndrome
ECCL: encephalocraniocutaneous lipomatosis.
